# Concentration-Dependent Decitabine Effects on Primary NK Cells Viability, Phenotype, and Function in the Absence of Obvious NK Cells Proliferation–Original Article

**DOI:** 10.3389/fphar.2021.755662

**Published:** 2021-10-25

**Authors:** Xiang Li, Min Zhang, Sisi Cai, Yaohui Wu, Yong You, Xianghong Wang, Li Wang

**Affiliations:** ^1^ Institution of Hematology, Union Hospital, Tongji Medical College, Huazhong University of Science and Technology, Wuhan, China; ^2^ Institution of Hematology, The Central Hospital of Wuhan, Tongji Medical College, Huazhong University of Science and Technology, Wuhan, China

**Keywords:** acute myeloid leukemia, natural killer cells, decitabine, p-ERK, P-STAT3

## Abstract

Acute myeloid leukemia (AML) cells can evade innate immune killing by modulating natural killer (NK) cells receptors and their cognate ligands in tumor cells, thus it may be possible to restore proper expression of immune receptors or ligands with immune sensitive drugs. Decitabine, as a hypomethylation agent, was approved for the treatment of AML and myelodysplastic syndrome. While clinical responses were contributed by epigenetic effects and the induction of cancer cell apoptosis, decitabine also has immune-mediated anti-tumor effects. After exposure to various concentration of decitabine for 24 h, the primary NK cells (AML-NK cells) cytotoxicity and receptor expression (NKG2D and NKp46) displayed parabola-shaped response, while U-shaped response was seen in cytokine release (IFN-γ and IL-10), and these effects were regulated by ERK and STAT3 phosphorylation level. Furthermore, AML-NK cells function displayed different response when the competitive MEK and STAT3 inhibitors applied respectively. Thus, we could conclude that the different dose of decitabine makes various effects on AML-NK cells function and receptors expression.

## Introduction

Acute myeloid leukemia (AML), is caused by hyperproliferation and/or impaired differentiation of myeloid precursor cells, mostly affecting older individuals at a median age of 67 years (Estey, 2007; Oran and Weisdorf, 2012). For many years, the standard treatment for AML, which consists of chemotherapeutic induction therapy, consisting of 3 days of topoisomerase inhibitor (e.g., daunorubicin) administration and 7 days of DNA synthesis inhibitor (e.g., cytarabine with or without etoposide) administration yields a remission rate of only 60% (Geiger and Rubnitz, 2015). Moreover, age and cytogenetics are important prognostic factors for patients with AML, and AML patients with a TP53 mutation or who are classified at high-risk groups respond poorly to traditional chemotherapy and had reduced overall survival relative to non-adverse AML patients. Allogeneic hematopoietic stem cell transplantation represents a potentially curative treatment for AML, but It does not eliminate the risk of relapse completely and it is counter-indicated in many elderly patients due to comorbidities or poor performance status. Thus, the identification of new treatment effective for AML, but spare many of the treatment-related risks of stem cell transplant would be a primary objective in this field.

Decitabine, one kind of hypomethylation agents, was FDA-approved in the treatment of patients with MDS and had a well-established palliative role in the treatment of patients with AML who are unlikely to benefit from standard induction chemotherapy due to advanced age or poor performance status (Yang et al., 2014; Gao et al., 2015). Notably, the benefits could also be achieved in patients with the TP53 mutation (Welch et al., 2016), whose prognosis was rather terrible. A 10-days course of decitabine has been shown to augment antibody-dependent-cell mediated cytotoxicity responses (Vasu et al., 2016). Additionally, treatment with decitabine for 28 days increases natural killer (NK) cell-mediated lysis of AML cells and increases expression of the killer cell lectin-like receptor K1 (KLRK1) ligand on tumor cells (Li et al., 2017; Tang et al., 2008). Hence, the clinical benefits of decitabine treatment may involve the combination of a direct cytotoxic effect on malignant cells and an indirect antitumor effect mediated through immunoregulation. Although several studies have examined decitabine dose optimization, there remains limited data regarding the direct effects of decitabine on the immune system (Gao et al., 2009; Schmiedel et al., 2011; Lisa et al., 2013; Sohlberg et al., 2015), especially on NK cells.

NK cells are critical mediators of the innate immune response, acting as the first line of defense against viral infection and the malignant transformation of normal cells. In patients with AML, NK cells are markedly reduced in number and show a dramatic impairment in cytotoxic efficacy due to down-regulation of NK cell-activating receptors [particularly KLRK1 and natural cytotoxicity triggering receptor 1 (NCR1) and 3], defective AML-NK synapse formation, and increased shedding of soluble ligand forms (Costello et al., 2002; Fauriat et al., 2007; Khaznadar et al., 2014; Lichtenegger et al., 2014). Therefore, normalization of NK cells function and numbers in AML patients may have important influence on patients recovery and prognosis (Kim et al., 2005; Larghero et al., 2007).

CD107a is a lysosome-related protein that relocates to the cell membrane when NK cells are activated and undergoing degranulation, during which time cytolytic granules are released into target cells. Thus, CD107a expression can serve as a surrogate for the cytotoxic potential of NK cells. The aim of the present study was to explore the effects of different-dose decitabine on primary NK cells function and the underlying mechanism of those effects.

## Materials and Methods

### Patient Characteristics

This study was carried out in accordance with the regulations of the Declaration of Helsinki. All enrolled patients provided written informed consent to participate. Peripheral blood samples were collected from 18 patients diagnosed with AML, who had not yet received treatment, from June 2016 to October 2017. Patients with acute promyelotic leukemia were excluded from this study. The participating patients’ characteristics are summarized in Table 1.

**TABLE 1:** The clinical characteristics of AML patients in our study.

	Sex	Age (year)	Diagnosis FAB subtypes	Blast cells (%)	Cytogenetic
1	M	76	M1	49	indefinite
2	M	65	M2	38	Low-risk
3	M	47	M1	23.5	Intermediate-risk
4	M	38	M1	33.6	Poor-risk
5	F	46	M2	36	Poor-risk
6	F	70	M5	44.5	Intermediate-risk
7	F	56	M2	39	Intermediate-risk
8	M	53	M2	49	Intermediate-risk
9	M	65	M5	33.5	Intermediate-risk
10	F	69	M5	66.2	Intermediate-risk
11	M	51	M4	50.5	Intermediate-risk
12	M	43	M4	51.5	Poor-risk
13	F	61	M2	46	Poor-risk
14	F	65	M2	40.6	Poor-risk
15	F	72	M2	39.2	Intermediate-risk
16	M	71	M4	43.2	Poor-risk
17	M	70	M5	45.2	Intermediate-risk
18	M	67	M4	35.1	Intermediate-risk

The diagnosis was according to the FAB classification, 3-M1, 7-M2, 4-M4, 4-M5.

The risk stratifying was according to the 2016 NCCN guidelines.

The median age was 54 years-old, 11 of 18 patients were men, the others were women.

The bone marrow blasts >30% was seen in almost all the patients.

### Cell Preparations and Cultures

Peripheral blood mononuclear cells were isolated from each patient’s blood sample by Ficoll lymphoprep density gradient centrifugation (BD science, Tianjin, China). NK cells were purified with a NK isolation kit (MiltenyiBiotec, Germany) to a ≥98% CD56 expression and <1% CD3 expression. Cell viability exceeded 90%, as determined by trypan blue staining. The separated NK cells were cultured in 6-well plates at a density of 5 × 10^5^ cells/ml in RPMI 1640 medium (GE Health Life Science, United States), supplemented with 15% of fetal bovine serum (Gibco Invitrogen), 100 U/ml of penicillin-streptomycin (Gibco Invitrogen), and 500 U/ml of interleukin-2 (peprotech, United States). K562 cells were grown in RPMI 1640 medium supplemented with 10% fetal calf serum (Zhejiang Tianhang Technology, Zhejiang, China) and 100 U/ml of penicillin-streptomycin (Gibco Invitrogen). Because NK cells were not cultured under continuous proliferation conditions and a longer culture time (>48 h) would induce widespread apoptosis of NK cells, we used a 24 h NK cells culture period.

### Application of Drugs to NK Cells in Culture

Decitabine (Sigma-Aldrich, St. Louis, MO) was dissolved in double-distilled water to a 5 mmol/L stock concentration, aliquoted for single use, and stored at −20°C. The competitive MEK (mitogen-activated protein kinase kinase) inhibitor U0126 (Medchem Express, United States) was applied to culture media at a concentration of 100 μmol/L. The STAT3 (signal transducer and activator of transcription) inhibitor S3I-201 (Medchem Express, United States) was added to culture media at a concentration of 50 μmol/L. Control groups were treated with only dimethyl sulfoxide and decitabine.

### Cell Viability Assays

Cell viability was determined with a cell counting kit-8 (CCK-8; Dojindo, Shanghai, China) according to the manufacturer’s instructions. Viability was determined from the intensity of optical absorption at 450 nm by a microplate reader (Synergy™2, Biotek, Winooski, VT). Annexin V-based apoptosis assays (keyGENBioTECH, Nanjing, China) were performed in accordance with the manufacturer’s instructions.

### Flow Cytometry

Expression of the NK cell receptors was evaluated by flow cytometry after exposure to various concentrations of decitabine for 24 h. Mouse anti-human PE-labeled CD56 (clone: HCD56), Percp-labeled CD3 (clone: HIT3a), FITC-labeled NKp46 (clone: 9E2), APC-labeled NKG2D (clone: 1D11), and CD158b (clone: DX27) were purchased from Biolegend (San Diego, CA). Each antibody was added to NK cell cultures and incubated for 30 min at 4°C. Cells were washed twice with phosphate-buffered saline before being subjected to assessment with a FACS Canto I machine (BD science, Franklin Lakes, NJ).

### Cytotoxicity Assay

After being exposed to experimentally indicated concentrations of decitabine for 24 h, NK cells cytotoxicity assays were performed as described in detail elsewhere (Gao et al., 2009). Briefly, NK cells and k562 target cells were mixed at an effector/target ratio of 2:1 (5 × 10^5^: 2.5 × 10^5^) in a total volume of 200 µl in a 96-well plate. After incubation for 2 h, 2 µl of 2 mmol/L monensin (Biolegend) were added and the cells were incubated for an additional 3.5 h. Finally, the cells were collected and stained with anti -CD56, -CD3, and -CD107a (clone: H4A3) antibodies for 30 min at 4°C before being submitted to analysis in a FACS Canto I machine (BD science).

### Determination of Cytokine Expression Levels

Concentrations of interferon gamma (IFN-γ) and interleukin 10 (IL-10) in culture supernatants were determined with an enzyme-linked immunosorbent assay (ELISA) kit (Invitrogen, United States) according to the manufacturer’s instructions. The detection limits of ELISA kits for IFN-γ and IL-10 are 1.6–100 pg/ml and 0.39–25 pg/ml, respectively.

### Western Blotting

Western blotting analyses were performed as previously described (Simar et al., 2014; Wang et al., 2015). Total soluble proteins were extracted with radio-immunoprecipitation assay lyses buffer (Applygen Technologies Inc., Beijing, China) containing phenylmethane sulfonyl fluoride (Sigma-Aldrich). Protein concentrations were determined with the BCA method; equal amounts of total protein were loaded on 10% denatured polyacrylamide gels and transferred electrophoretically to nitrocellulose membranes (Hybond). The blotted membranes were blocked with 5% (w/v) nonfat derived milk in Tris-buffered saline for 1 h at room temperature and then were incubated with primary antibodies against β-actin, ERK (extracellular signal-regulated kinase), STAT3 (signal transducer and activator of transcription), phosphorylated (*p*)-ERK, and p-STAT3 (1:1,500, Cell Signaling Technology) for 16 h at 4°C. After incubation with IRDye 800 CW-conjugated goat anti-rabbit IgG antibodies, the labeled bands were visualized and quantified with an Odyssey Infrared Imaging system (LI-COR. Bioscience).

### Quantitative Reverse Transcriptase-Polymerase Chain Reaction

Total RNA from NK cells was isolated using RNA isolation kit (Roche, Mannheim, Germany). Synthesis of cDNA was performed using the Superscript™ II reverse transcriptase kit (Invitrogen, Karlsruhe, Germany). For quantitative RT-PCR analysis, the human SOCS3 Lightcycler™-primer set (Search LC, Heidelberg, Germany)was used according to manufacturer’s instructions.

### Statistical Analysis

Quantitative results are expressed as the means ± standard errors of the mean. Inter-group differences were detected with one-way analyses of variance (ANOVAs) and Student-Newman-Keuls tests. *p* values of less than 0.05 were considered to be significant. All results were analyzed in SPSS 13.0 software (SPSS, Chicago, IL).

## Results

### The Primary NK Cells Viability Declined With Increasing Decitabine Concentrations

**FIGURE 1:**
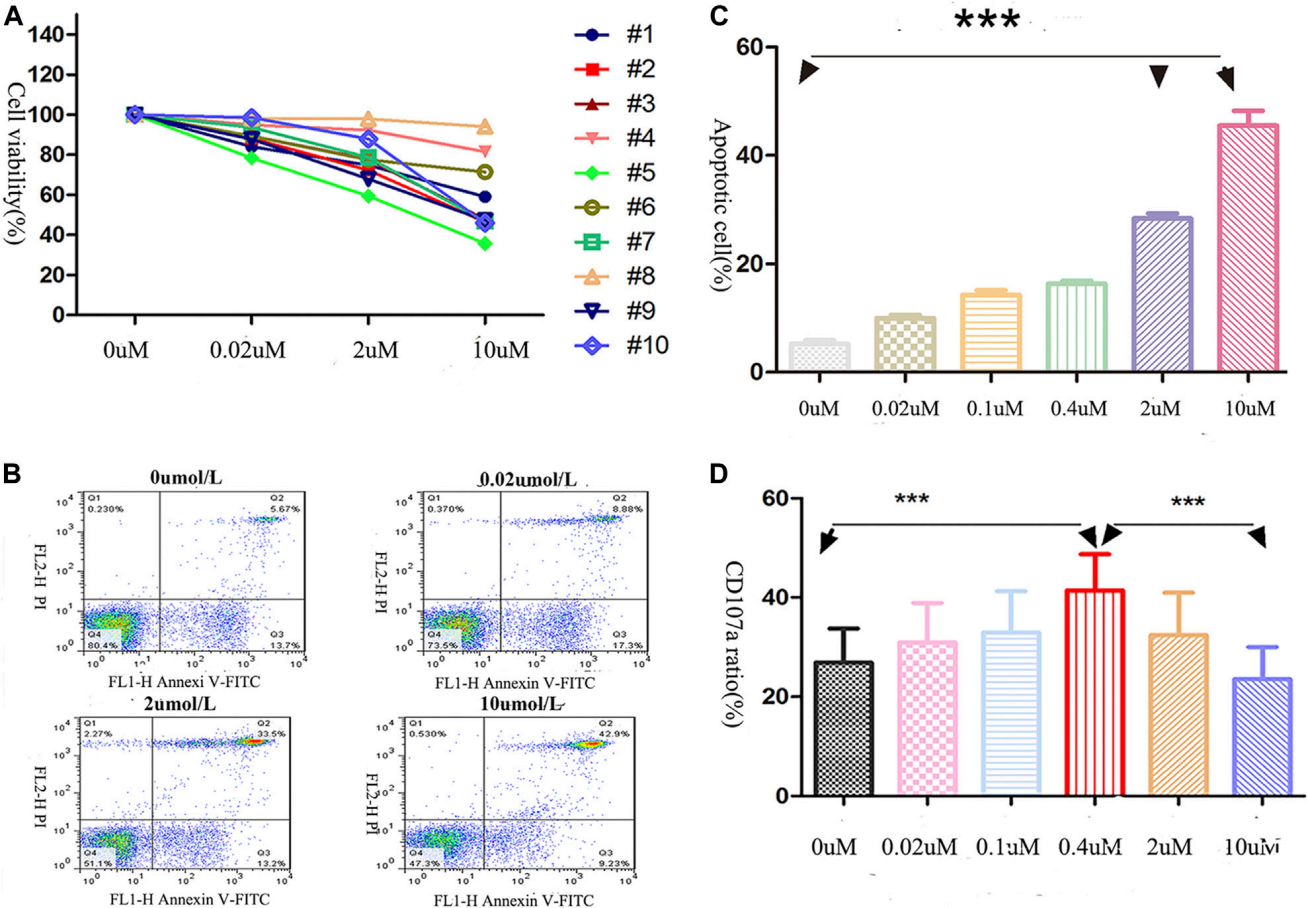
Effects of decitabine exposure on primary NK cells viability and cytotoxicity. **(A)** NK cells viability was assessed by CCK-8 assay. After being cultured for 24 h (40,000 cells/well in 96-well plates), cells were incubated with CCK-8 solution for 4 h **(B,C)** The dead NK cells were visualized with annexin V/PI double-staining (performed in triplicate): 0 μmol/L, 5.67%; 0.02 μmol/L, 8.88%; 2 μmol/L, 33.55%; 10 μmol/L, 42.9%. **(C)** The highest decitabine concentration group (10 μmol/L) had more dead NK cells than other groups (*n* = 3, *p* < 0.05). The statistical difference existed between 0 and 2 uM treatment groups, 0 and 10 uM treated groups. **(D)** NK cells cytotoxicity was evaluated by CD107a expression on the membrane surface (effector/target = 2:1), ***p < 0.0001 (*n* = 5/group).

The viability of NK cells (obtained from 10 AML patients, AML-NK cells) decreased after exposure to decitabine for 24 h (Figure 1A) and it was induced by direct cell death (dead cells rate rose sharply as the concentration of decitabine increased (Figure 1B). The dead cells rate was significantly greater in the 10 umol/L decitabine concentration group than other concentration groups (Figure 1C).

### Concentration-Dependent Decitabine Effects on Primary NK Cells Cytotoxicity

As shown in Figure 1D, after the AML-NK cells exposed to various concentration of decitabine for 24 h, we observed a parabolic concentration-response curve for decitabine-induced CD107a expression on AML-NK cells, and the peak effect was observed in the 0.4 μmol/L concentration group.

### The Influence of Clinical Characteristics on Primary NK Cells Cytotoxicity

**FIGURE 2:**
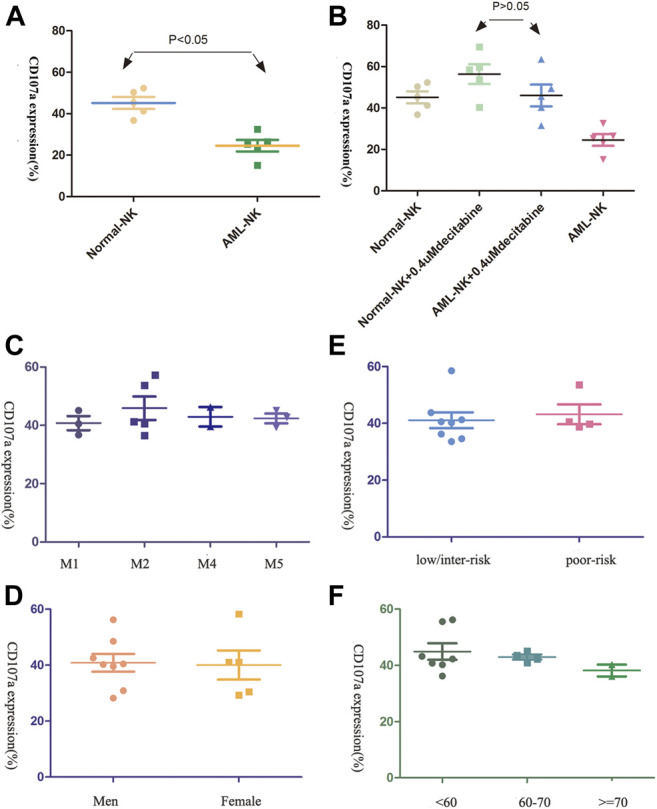
The cytotoxicity of primary NK cells was seldom influenced by patients clinical characteristics **(A,B)** The cytotoxicity of AML-NK cells was impaired relative to that of Normal-NK cells (**p < 0.05). Cytotoxicity of AML-NK cells was nearly equal to that of Normal-NK cells when treated with 0.4 μmol/L decitabine. **(C,D)** Cytotoxicity of AML-NK cells did not differ among FAB subgroups (M1, M2, M4, M5), gender(the male to female ratio of donor patients was 11:7) **(E)** There was no obvious difference between low/ intermediate-risk and high/poor-risk patients when treated with 0.4 μmol/L decitabine. **(F)** There was no obvious difference among patients <60 years old, patients 60–69 years old, and patients ≥70 years old when treated with 0.4 μmol/L decitabine.

As shown in Figure 2A, the AML-NK cells were found to have impaired cytotoxicity compared to NK cells obtained from healthy donors (Normal-NK cells), but the cytotoxicity of AML-NK cells nearly equal to the Normal-NK cells when exposure to 0.4uM decitabine (Figure 2B, *p* > 0.05). In addition, There was no prominent difference when compared the AML-NK cells cytotoxicity among various sub-group, which implied that the AML-NK cells cytotoxicity was independent of FAB subtype, patient age, gender, and risk stratification (Figures 2C–F).

### Concentration-Dependent Decitabine Effects on Primary NK Cells Receptor Expression

**FIGURE 3:**
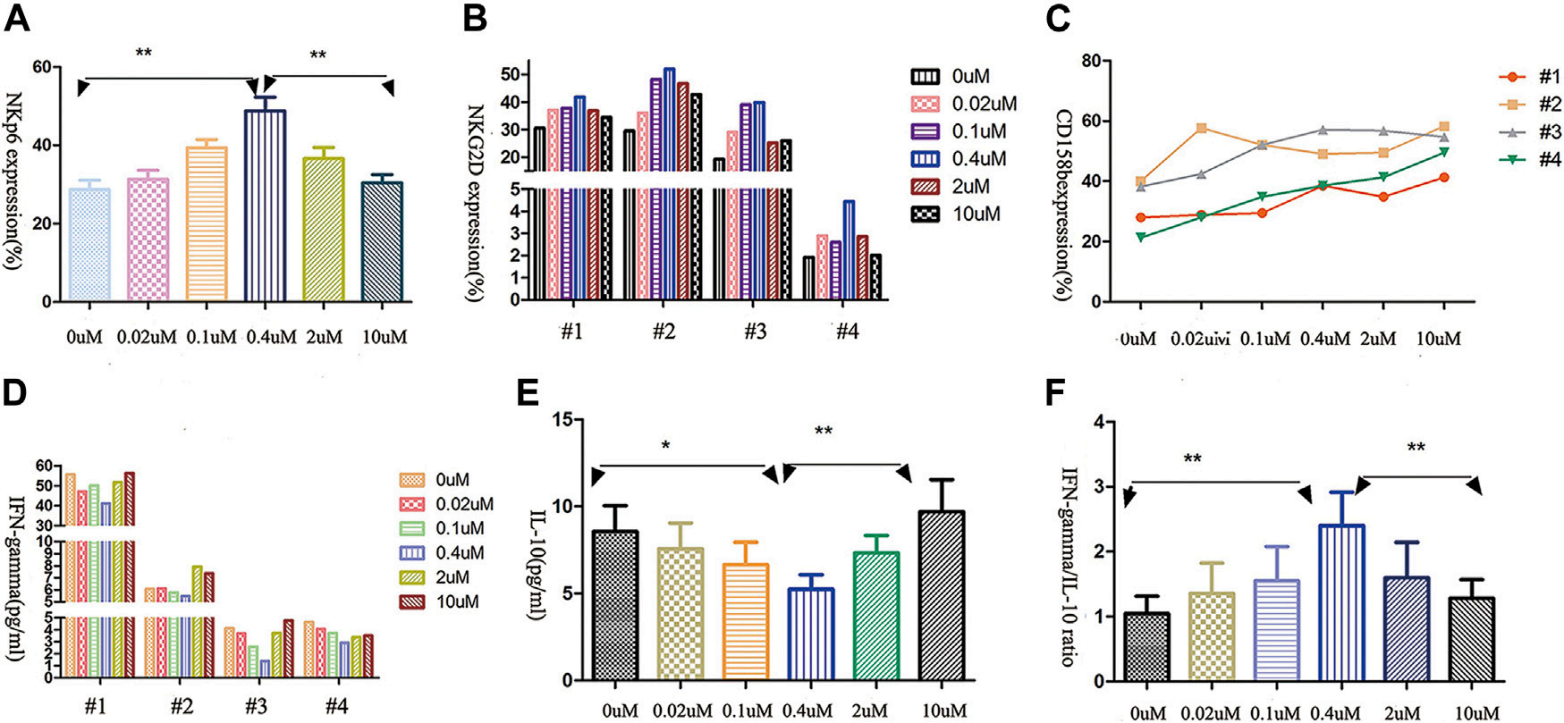
The effects of decitabine exposure on primary NK cells receptor expression and cytokine release. **(A,B)** The expression of NK cells-activating receptors NKG2D and NKp46 showed parabola-shaped concentration-dependent response to decitabine exposure (*n* = 4; *p* = 0.0038). **(C)** The expression of inhibitory receptor CD158a increased with the increasing concentrations of decitabine **(D,E)** The release of IFN-γand IL-10 both had U-shaped concentration-dependent responses, with nadirs at 0.4 μmol/L treatment group. **(F)** The ratio of IFN-γto IL-10 showed parabolic response, peak value at 0.4 μmol/L treatment groups (*n* = 4, *p* = 0.0034).

Since the function of NK cells is regulated by a balance between several signals, we assessed the expression of activating and inhibitory killer-cell immunoglobulin-like receptors (KIRs) on AML-NK cells. The major NK cells activating receptors were NKp46 and NKG2D.CD158b was the common inhibitory receptors in NK cells, which plays prominent roles in immune tolerance. As shown in Figures 3A,B, we observed a parabola-shaped curve of the activating receptors NKG2D and NKp46 expression on AML-NK cells surface after exposure to a range of concentrations of decitabine for 24 h (0–10 μmol/L) and the NKp46 expression levels differed significantly among different groups (Figure 3A, *p* = 0.0038). Whereas, the CD158b expression level increased consistently with rising concentrations of decitabine (Figure 3C).

### Concentration-Dependent Decitabine Effects on Cytokine Release

IFN-γ (a positive immunomodulatory factor) and IL-10 (a negative immunomodulatory factor) both had similar U-shaped curve when exposured to increasing concentrations of decitabine for 24 h (Figures 3D,E). Notably, due to IL-10 concentration increasing faster, the ratio of IFN-γ to IL-10 displayed a parabolic-shaped response, and the maximum ratio value was observed at the 0.4 μmol/L concentration of decitabine (Figure 3F).

### The Opposite Decitabine Effects on Phosphorylation Levels of ERK and STAT3

**FIGURE 4:**
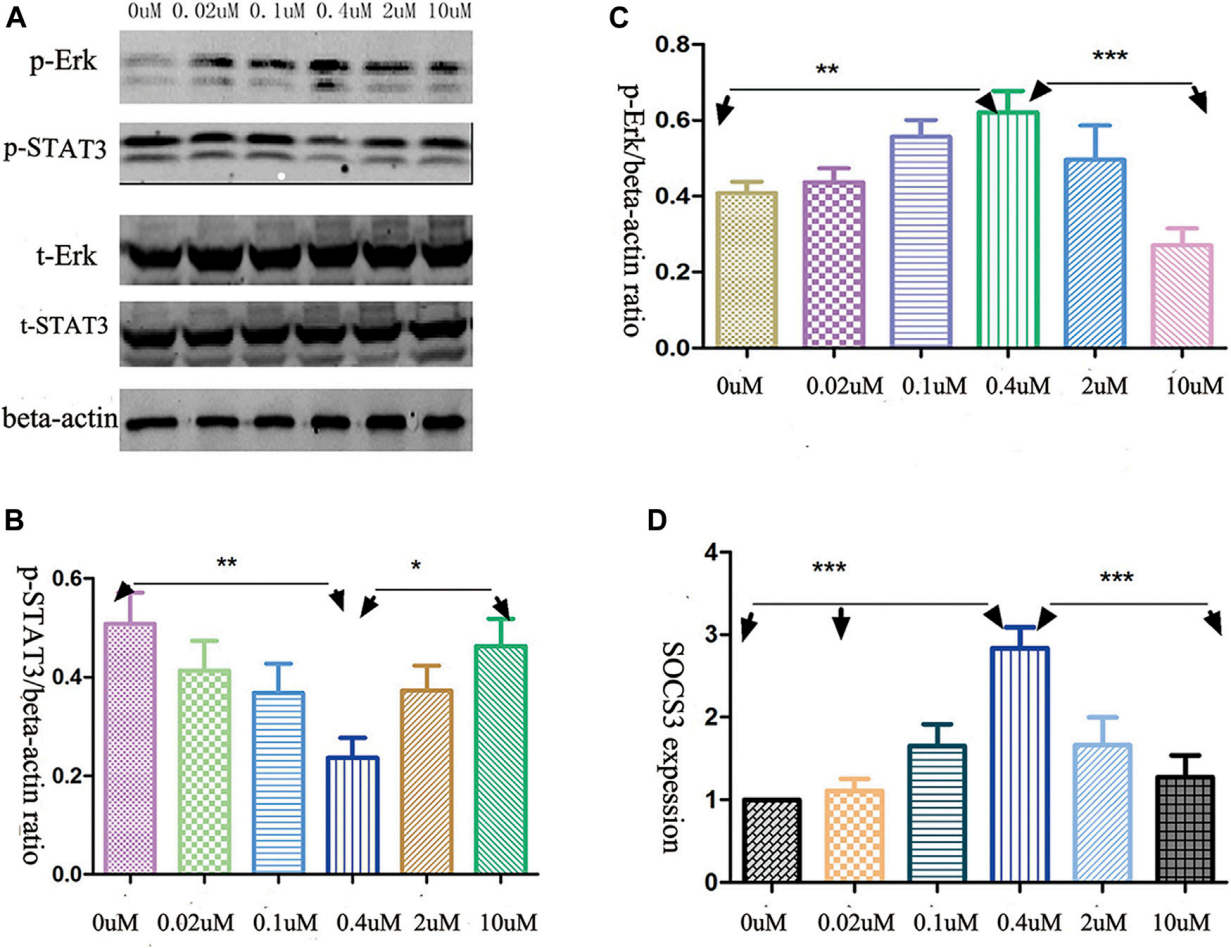
Decitabine effects on signaling pathways. **(A)** Western blot analysis revealed the opposite effects of decitabine exposure on p-ERK and p-STAT3 levels **(B,C)** Quantitative analyses of relative p-STAT3 and p-ERK levels in NK cells, devired from seven patients, normalized to β-actin (*n* = 7, *p = 0.03, **p = 0.0011, ***p < 0.0001). **(D)** qRT-PCR analysis of transcript levels of SOCS3, an endogeneous feedback inhibitor of STAT3 signaling pathway (*n* = 6, *p* < 0.001).

The levels of phosphorylated ERK and STAT3 had been shown to be related to NK cells cytotoxicity and cytokine release (Vivier et al., 2004; Cacalano, 2016). Therefore, we investigated the phosphorylation level of ERK and STAT3 in AML-NK cells when exposed to various doses of decitabine for 24 h. Western blot analysis showed that the opposite response was found in the phosphorylation level of ERK and STAT3 (Figure 4A) when exposured AML-NK cells to decitabine for 24 h. A follow-up experiment with AML-NK cells from a subset of seven patients replicated the same pattern of results, as seen in Figure 4A (Figures 4B,C).

### Decitabine Exposure Alters SOCS3(STAT3 Signaling Pathway Regulatory Factor) Expression

A quantitative RT-PCR experiment showed that the mRNA expression of SOCS3 exhibited parabola-shaped response to 24 h decitabine exposure (Figure 4D). The SOCS3 expression in the 0.4 μmol/L treatment group differed significantly from that in the other groups (Figure 4D).

### Inhibitors of MAPK and STAT3 Signaling Pathway Affect AML-NK Cells Cytotoxicity, NK Cells Receptor Expression, and Cytokine Release

**FIGURE 5:**
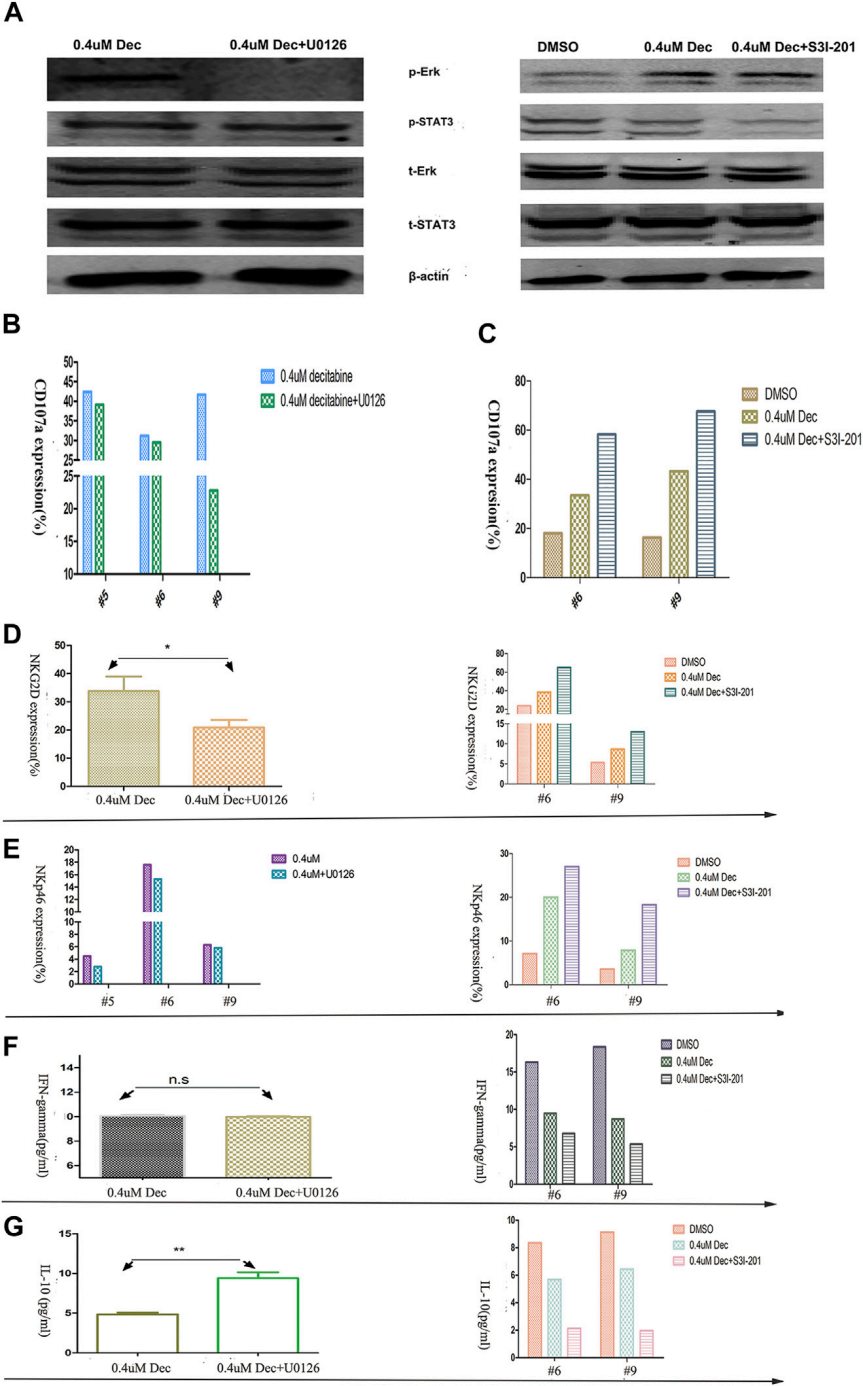
Effects of ERK and STAT3 phosphorylation inhibitors on primary NK cells cytotoxicity, activating KIR expression, and cytokine release. **(A)** The response of p-ERK and p-STAT3 levels to U0126 (100 μmol/L) and S3I-201 (50 μmol/L) exposure. In the case of U0126, NK cells were pretreated, centrifuged, and then exposed to decitabine (pretreatment omitted control group). In the case of S3I-201, NK cells were co- cultured with S3I-201 and decitabine (S3I-201 omitted control group) **(B,C)** U0126/S3I-201 (#5, #6, #9) effects on NK cells cytotoxicity, as indexed by CD107a expression **(D,E)** U0126 (100 μmol/L) decreased and S3I-201 (50 μmol/L) increased the expression of NK cell-activating receptors (NKG2D and NKp46) (*n* = 3, *p* = 0.027) **(F,G)** U0126 versus S3I-201 had differing effects on cytokine release (*n* = 3, *p* = 0.0071).

Western blot analysis showed that 100 μmol/L U0126 fully inhibited the ERK phosphorylation, whereas 50 μmol/L S3I-201 partially inhibited STAT3 phosphorylation (Figure 5A). Pretreatment of AML-NK cells with the MAPK signaling pathway inhibitor-U0126 for 1 h had a weak to negligible cytotoxicity-reducing effect (Figure 5B) and reduced the expression of NK cells-activating receptors (NKp46 and NKG2D), with the latter decreasing more rapidly (Figures 5D,E). On the other hand, co-treatment with the STAT3 signaling pathway inhibitor S3I-201 and 0.4 umol/L decitabine for 24 h had a dramatic cytotoxicity-enhancing influence on AML-NK cells (Figure 5C), and resulted in markedly increased expression levels of NKp46 and NKG2D (Figures 5D,E).

Finally, ELISA experiments showed that co-treatment S3I-201 with 0.4 umol/L decitabine reduced IFN-γ release significantly (Figure 5F), whereas pretreatment with the U0126 did not affect IFN-γ release of AML-NK cells. Meanwhile, we observed increased IL-10 release in AML-NK cells when pretreated with U0126 and markedly reduced IL-10 release when co-treated with S3I-201 for 24 h (Figure 5G).

## Discussion

NK cells are bone marrow-derived lymphocytes that constitute a key frontline defense against a range of hazardous conditions, including viral infection and tumor transformation (Biron et al., 1999; Cerwenka and Lanier, 2001). NK cells differ from B and T-lymphocytes in that rather than relying on a richness of antigen-specific receptors, NK cells activation is induced by a lack of recognition of major histocompatibility complex class I molecules by KIRs together with recognition of stress-related ligands binding their activating receptors (Höglund and Brodin, 2010). Because NK cells are critical for leukemia treatment effectiveness (Kim et al., 2005; Larghero et al., 2007), there has been great interest in developing autologous or allogeneic NK cells engrafts with the goal of augmenting immune destruction of leukemia cells by stimulating (or preventing the suppression of NK cell cytotoxicity (Ljunggren and Malmberg, 2007; Terme et al., 2008).

Decitabine, which is a cytosine analogue, has a direct cytotoxic effect at higher doses and a hypomethylation effect at lower doses (Stresemann and Lyko, 2008). It is used in the management of patients with hematological malignancies, especially for elderly patients who cannot tolerate standard or intensive chemotherapy due to their age-related frailty or poor performance status. Several lines of evidence suggest that hypomethylation agents can also influence the immune system (Wang et al., 2015; Vasu et al., 2016; Li et al., 2017). For example, it was reported that the methylation level of NK cells was increased in the context of type 2 diabetes, and the heightened methylation level had been shown to lead to insulin resistance (Simar et al., 2014), consistent with a potential indirect effect on the immune system, especially on NK cells. Different from the results reported by Kopp et al. (Schmiedel et al., 2011; Lisa et al., 2013), our results showed that the cytotoxicity of AML-NK cells presented the “parabolic-shaped” response after 24 h decitabine exposure. The explanation for this difference between our data and Kopp et al.’s may be that: 1) the different sources of NK cells. that is: the primary NK cells used in our study were from AML patients while those in Kopp’s study were from the healthy donors; 2) there was no feeder cells in our culture conditions, while the feeder cells were used in Kopp’s study.

Chan et al. (2003) demonstrated that KIR expression was regulated at the transcription level by epigenetic changes in complex and synchronized fashion. Here, we observed gradually increasing expression of CD158b with the increasing concentrations of decitabine, consistent with the previous data (Schmiedel et al., 2011; Lisa et al., 2013), but the parabolic-shaped response of the NKp46 and NKG2D expression was displayed with the increasing concentrations of decitabine.The difference between the expression profiles of activating and inhibitory receptors in our study may be due to the unaffected status of CD158b expression on primary human NK cells in resting and cell-division states.

With respect to the effects of decitabine on cytokine release, we found that IFN-γ and IL-10 release both displayed U-shaped response, the lowest release levels in the 0.4 μmol/L concentration group. Surprisingly, the IFN-γ to IL-10 ratio exhibited a parabola-shaped response, with the maximum value in the 0.4 μmol/L concentration group, implying that IL-10 release increased faster than IFN-γ release, yielding a dissociation of the curves in the 0.4–10 μmol/L concentration range. So, based on our results, we can make three suppositions: Firstly, we posit that decitabine can make different effects on primary NK cells function depending on the dose given. Secondly, our findings suggest that the lower doses of decitabine can enhance the primary NK cells function, whereas higher doses of decitabine impair the primary NK cells function through down-regulation of NK activating KIRs (NKG2D and NKp46) and promotion of the release of suppressive immunomodulators, such as IL-10. Thirdly, we believe it will be important to conduct detailed studies in the future to clarify the optimal dose of decitabine for enhancing NK cells function with the goal of achieving a clinically significant benefit with minimal adverse secondary effects.

The STAT3 signaling pathway is controlled by several endogenous feedback inhibitors, including suppressor of cytokine signaling 3 (SOCS3) (Kubo et al., 2003), which is regulated by epigenetic mechanisms—namely CpG methylation and acetylation of CpG islands in the *SOCS3* promoter. (Croker et al., 2003; Kim et al., 2015). In our study, the parabola-shaped response was seen in SOCS3 expression when exposed to various doses of decitabine for 24 h. So the different response of ERK and STAT3 phosphorylation level may be due to SOCS3 effects (Kim et al., 2015).

Consistent with prior studies (Kortylewski et al., 2005; Gotthardt et al., 2014), we found that AML-NK cells cytotoxicity and the expression of activating receptors both declined when the cells were pretreated with U0126, but opposed effects were seen when S3I-201 was added to decitabine. Conversely, Hoelbl-Kovacic (Putz et al., 2014) found that STAT3 knock-out in tumor cell lines can weaken the function of NK cells. The discrepancies between these studies may be due to the different kinds of study objects (hematopoietic cells, NK cells, and tumor cell lines). Furthermore, tumor cell-produced cytokines have been shown to induce higher levels of STAT3 phosphorylation in infiltrating immune cells while suppressing immune cell activity (Cheng et al., 2003; Wang et al., 2004). Therefore, it is possible that the phosphorylation level of STAT3 may have a negative modulatory effect on NK cells cytotoxicity and release of cytokines in patients with cancers. Currently, specific STAT3 inhibitors and *STAT3* antisense oligonucleotides are undergoing biosafety clinical trials (NSC00955812, NCT00696176, and NTC01563302). It will be of great interest to learn whether STAT3 inhibition may thus have dual anti-cancer benefits.

Varying doses of decitabine have been shown to produce differing plasma concentrations in patients with hematological malignancies (van Groeningen et al., 1986; Cashen et al., 2006; Cashen et al., 2010). For example, 20 mg/m^2^ of decitabine (a common dose given to patients with leukemia or myelodysplastic syndrome) can produce a plasma concentration of approximately 1.25 μmol/L (Cashen et al., 2010). In our study, the primary NK cells function was maximal at a relatively low concentration of decitabine (0.4 μmol/L). Thus, we believe that decitabine dosage and treatment time should be further explored to achieve an optimal clinical effect.

In conclusion, our findings support the consideration of the development of future therapeutic strategies involving decitabine. Firstly, in addition to direct effects on tumor cells, decitabine may be able to promote primary NK cells function. Secondly, decitabine could potentially be used to augment the ability of primary or migrated NK cells to attack leukemia cells. We did not detect NK cells proliferation after exposure to a range of decitabine concentration, indicating that the presently observed NK cells responses to decitabine cannot be attributed to cell division-dependent methylation. Because some researchers have reported findings suggesting that hypomethylation agent effects on NK cells function depend on transcription modulation (Fauriat et al., 2007; Schmiedel et al., 2011), future studies should examine whether the presently observed effects of decitabine are mediated by decitabine-induced transcription modulation and, if so, whether the modulation is non-specific or targeted to specific genes. Currently, there is a paucity of data related to the effects of hypomethylation agents on human immunity. The present work thus provides information that contributes to filling this gap in knowledge, and subsequent studies involving more cases are needed to clarity the mechanisms underlying decitabine effects on anti-cancer immune responses.

## Data Availability

The original contributions presented in the study are included in the article/Supplementary Material, further inquiries can be directed to the corresponding author.
